# Gold Nanopost-Shell Arrays Fabricated by Nanoimprint Lithography as a Flexible Plasmonic Sensing Platform

**DOI:** 10.3390/nano9111519

**Published:** 2019-10-25

**Authors:** Cosmin Farcau, Daniel Marconi, Alia Colniță, Ioana Brezeștean, Lucian Barbu-Tudoran

**Affiliations:** 1 National Institute for Research and Development of Isotopic and Molecular Technologies, 67-103 Donat Str., 400293 Cluj-Napoca, Romania; daniel.marconi@itim-cj.ro (D.M.); alia.ungurean@itim-cj.ro (A.C.); ioana.brezestean@itim-cj.ro (I.B.); lucian.barbu@itim-cj.ro (L.B.-T.); 2 Institute for Interdisciplinary Research in Bio-Nano-Sciences, Babes-Bolyai University, 42 T. Laurian, 400271 Cluj-Napoca, Romania; 3 Faculty of Physics, Babes-Bolyai University, 1 M. Kogalniceanu, 400084 Cluj-Napoca, Romania; 4 Electronic Microscopy Laboratory and Department of Molecular Biology and Biotechnology, Babeș-Bolyai University, 44 George Bilascu Street, 400015 Cluj-Napoca, Romania

**Keywords:** surface plasmon, sensors, nanoposts, nanopillars, nanoimprint lithography, nanofabrication, finite-difference time-domain (FDTD) simulation

## Abstract

Plasmonic noble metal nanostructured films have a huge potential for the development of efficient, tunable, miniaturized optical sensors. Herein, we report on the fabrication and characterization of gold-coated nanopost arrays, their use as refractometric sensors, and their optimization through photonics simulations. Monolithic square nanopost arrays having different period and nanopost size are fabricated by nanoimprint lithography on polymer foils, and sputter-coated by gold films. The reflectivity of these gold nanopost-shell arrays present dips in the visible range, which are efficient for refractometric sensing. By finite-difference time-domain (FDTD) simulations we reproduce the experimental spectra, describe the electric fields distribution around the nanopost-shells, and then explain their good sensitivity, around 450 nm/RIU. Furthermore, we determine by simulations the influence of several geometrical parameters, such as array period, nanopost width, gold film thickness, and nanopost side coverage on both reflectivity spectra and sensing capabilities. Fully coated nanoposts provide an extremely deep reflectivity minimum, approaching zero, which makes the relative reflectivity change extremely high, more than two orders of magnitude higher than for partially coated nanoposts. These results contribute to the understanding of the plasmonic properties of metal coated nanopost arrays, and to the development of efficient platforms for sensing and other surface plasmon based applications.

## 1. Introduction

Optical sensing based on surface plasmon resonances in noble metal nanostructures (isolated particles, particle arrays, and films) is receiving a lot of interest nowadays [1,2]. Sensing with surface plasmons on flat gold films deposited on a prism surface, the so-called Kretschmann geometry, is, since many years, a well-established commercially available technology [3]. The technique relies on the sensitivity of surface plasmon modes to changes in the refractive index near the gold surface, and it thus allows the detection of these changes in real time and without labelling. However, the more recently developed nanostructured plasmonic sensing platforms have an optical response which can be tuned to the desired wavelength range, they can be integrated in small portable devices, and their sensitivity allows the development of a wide range of sensing applications. The sensing of pollutants, such as pesticides [4] or heavy metal ions [5], or biomarkers relevant to health and medicine [6] were recently demonstrated. Although colloidal plasmonic nanoparticles (in liquid suspensions) are very effective in many applications, nanostructured plasmonic crystals supported by solid substrates, due to their inherent morphology, can access a wider range of applications. 

In recent years, flexible polymer-based substrates have been proposed for developing new optical sensors with enhanced performance, due to their ability to undergo large mechanical deformation while keeping topography features intact, affordability, and amenity to mass production [7]. Polydimethylsiloxane (PDMS), poly(methylmethacrylate) (PMMA), polypropylene (PP), polyimide (PI), polystyrene (PS), and polyethyleneterephthalate (PET) have been successfully used as base support for fabricating nanostructures [8,9,10].

Plasmonic crystals can be fabricated in a wide range of sizes and shapes, out of gold or silver, which are up to now the most exploited plasmonic materials. A broad palette of methods to fabricate plasmonic nanostructures with regular surface patterns were proposed, such as conventional photolithography [11], electron-beam lithography [12], nanoimprint lithography [13], or colloidal lithography [14], to name a few. 

Nanoimprint lithography (NIL) is a manufacturing technology with potential to be used as a key nanolithography process in future integrated circuits and integrated optics [15]. NIL can be viewed as a micromolding process in which the printed features are defined by the topography of a template [16] and consists in the physical deformation of a thin resist film using a hard mold at high temperature and pressure. Since NIL is not based on modification of resist chemical structure by radiation, the resolution of the imprinted features is immune to many factors that limit the resolution of conventional lithography, such as wave diffraction, scattering and interference in the resist, backscattering from the substrate, and the chemistry of the resist and developer [17]. Due to the low-cost system setup, large-scale fabrication, high throughput capability, and sub-10 nm resolution, NIL has been flagged as a strong candidate for next generation lithography techniques in the next decade and beyond [18,19].

In the last few years, NIL has gained popularity in the fabrication of photonic and electronic products (such as photonic crystals, optical lenses, and optical grating especially in the field of flexible thin films), where the incorporation of nanopatterns upgraded their performances or functions [20,21]. The capabilities of NIL to prepare high-density nanostructures over large areas faster and with lower costs than focused ion beam lithography and electron beam lithography made it highly compatible with localized surface plasmon resonance (LSPR)-based applications [22]. Lucas et al. [13] showed a very powerful and flexible approach to fabricate large-area, uniformly-oriented noble metal nanoparticles arrays with a complete control of the plasmonic response using NIL and one-dimensional gratings. Large areas of gold nanodisks were fabricated on glass substrates by NIL and used for the plasmonic sensing of streptavidin [22] and as clinical LSPR immunoassay sensor for the detection of the prostate-specific antigen [23]. High-density arrays of gold nanorods and nanodisks with excellent stability, signal strength, and resolution were fabricated by Krishnamoorthy et al. [24] using a combination of NIL and copolymer lithography, with applications in surface-enhanced Raman scattering (SERS) and sensing. An innovative, three dimensional (3D) platform based on a gold nanodisk array supported on resist nanopillars with a perforated metal layer underneath for chemical and biomedical sensing was fabricated by thermal NIL [25]. Recently, Liu et al. [26] reported the use of NIL and anodic aluminum oxide (AAO) template to fabricate plasmonic nanopillars on plastic film with tunable optical properties, while Li et al. [27] fabricated a 3D plasmonic nanoantenna array for large-area and high-performance SERS. The NIL patterning of flexible, plastic substrates was used by Kumari et al. [28] to design a simple SPR based refractive index sensor. High-quality, gold-coated nanodome arrays were also successfully fabricated using NIL [29]. Periodic gold nanohole arrays fabricated by NIL was incorporated into a surface plasmon resonance (SPR) sensor based on enhanced optical transmission through sub-wavelength nanohole arrays [30,31]. Plasmonic arrays of elliptical nanoholes for dual-wavelength, dual-polarization refractive index sensing were patterned using a novel approach combining nanoimprint lithography into a hard mask followed by transfer of the patterns into the underlying gold layer by dry etching [32]. Recently, Zhou et al. [33] built up a plasmonic immunochip platform based on plasmonic gold microcave arrays by combining soft nanoimprint lithography, microfluidics, antibody functionalization, and mobile fiber spectrometry. Thus, the well-aligned arrays of nanopillars/nanodomes/nanodisks with perpendicular orientations to the substrate offer a high optical tunability by changing period, size, and shape of the unit nanostructures, and could become the main building blocks for new optical devices with promising potential applications.

In this paper we present results on the fabrication of square nanopost arrays coated by gold films, their optical and morphological characterization, and their exploitation in refractometric sensing. Nanoimprint lithography is applied to a polymer foil, in order to obtain fully flexible, monolithic nanopost arrays, which are then sputter-coated by gold. The morphology of these gold nanopost-shell arrays (NPSA) is analyzed by scanning electron microscopy. The reflectivity spectra are then measured in air, water, and water-glycerol mixtures. Finite-difference time-domain simulations are then employed to first reproduce the experimental reflectivity spectra, and then to analyze the electric fields around the gold nanopost-shells. Finally we study by simulations how a range of geometrical parameters, such as array period, nanopost width, side gold film thickness, and nanopost side coverage affect the reflectivity spectra and the sensing efficiency of the gold nanopost-shell arrays.

## 2. Materials and Methods

### 2.1. Fabrication of Flexible Polymer Nanopost Arrays

The nanopost arrays used to create the Au nanopost-shells were fabricated using Obducat EITRE^®^3 NIL equipment (Obducat AB, Lund, Sweden). In our experiments we used a 3″ silicon mold (NIL Technologies, ApS, Kongens Lyngby, Denmark) with periodic arrays of two types of 500 nm depth nanoholes (300 and 400 nm period). The mold consists of a silicon substrate treated with an antiadhesive layer of perfluorodecyltrichlorosilane to prevent sticking. The nanopatterning of the mold was done with 100 kV electron beam lithography with lateral and vertical tolerances of ±15%.

**Figure 1:**
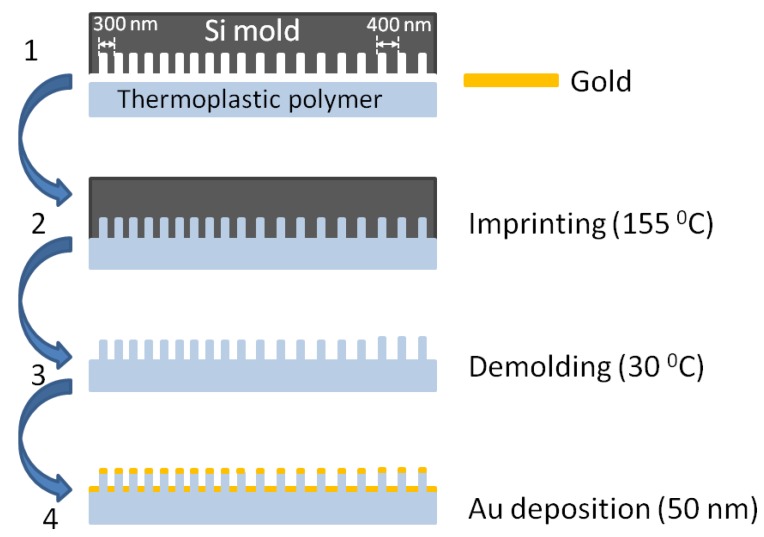
Schematic steps of the nanoimprint lithography process used for fabricating gold nanopost-shell arrays.

Figure 1 (Steps 1 to 3) presents the nanoimprint process steps on the flexible, thermoplastic substrate. The nanostructured, hard mold is pressed into the thin thermoplastic substrate. The thermoplastic substrate is deformed readily by the mold when heated above its glass transition temperature. During this step, the polymer behaves as a viscous liquid and can flow under applied pressure, thereby conforming to the mold. After the resist is cooled below its glass transition temperature, the mold is removed and the substrate solidification process occurs. The nanoholes arrays were transferred from the Si mold onto an IPS^®^ (Obducat AB, Lund, Sweden) flexible and transparent substrate (glass transition temperature *T_g_* ≈ 140 °C), having a thickness of 500 μm and a refractive index of 1.53, using thermal NIL. The maximum temperature of the imprinting process was 155 °C for 60 s and the maximum imprinting pressure was maintained at 40 bar. The cooling of the mold/plastic system took place at several intermediate pressures and temperatures down to 30 °C.

A 55 nm thick gold (Au) film was deposited (Step 4 in Figure 1) on the patterned plastic using Q150R PLUS sputter coater equipment (Quorum Technologies Ltd., Lewes, UK) from a disc-style Au target (57 mm diameter, 0.1 mm thickness) by a 25 mA direct current (DC) magnetron sputtering process.The DC process was carried out in a vacuum chamber at a base pressure of 10^−3^ mbar using argon (Ar) gas (99.999% purity), with a 25 mA sputter current by using DC power supply and a deposition rate of 2.0 to 2.5 nm/min. The deposition chamber was filled with pure Ar gas for target cleaning. The deposition was performed at a fixed substrate to source distance of 27 mm with the substrate rotating at a rate of 10 rpm. The substrate’s temperature was maintained at room temperature. The two types of samples, fabricated based on the 300 and 400 nm patterns, will be denoted as P300 and P400, respectively.

### 2.2. Scanning Electron Microscopy

The scanning electron microscopy (SEM) analysis was achieved by using an ultra-high resolution SEM Hitachi 8230 (Tokyo, Japan) system operated in high vacuum conditions. High resolution SEM images acquired at low landing voltage were assessed without destroying the sample. 

### 2.3. Optical Reflectivity Measurements

Optical images and reflectivity (*R*) spectra were recorded through the microscope of a Witec Alpha 300R system (WITec GmbH, Ulm, Germany), equipped with a very low numerical aperture (NA = 0.1) objective. This ensures both that a uniform clean area of the sample can be selected and analyzed, and that the incident angle is kept close to normal incidence. Light was collected by an optical fiber and directed to an Ocean Optics USB4000 (Ocean Optics Inc., Largo, FL, USA) spectrophotometer. Measurements were performed both in air and water–glycerol liquid mixtures. For analyzing the reflectivity in liquids, a cell was custom-built in a plexiglass plate, shaped as a rectangular well, 1 mm deep. The sample is placed at the bottom of this well, covered by liquid and topped by a microscope glass slide. Light is delivered to and collected from the sample surface by the microscope objective, after passing through the top glass slide and the liquid. The reflectivity was measured in solutions with progressively increasing refractive index, from 1.333 to 1.387 obtained by mixing water and glycerol (Sigma-Aldrich Corporation, St. Louis, MO, USA) in different proportions, from 100/0 to 60/40 water/glycerol.

### 2.4. FDTD Simulations

**Figure 2:**
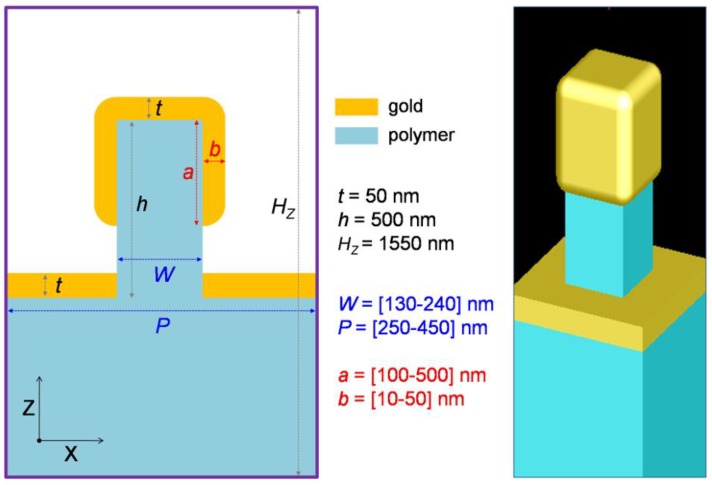
Geometry and parameters of the setup used for FDTD simulations: *P*—period of the array and width of the simulation cell along X and Y directions, *H_Z_*—vertical dimension of the simulation cell, *h*—height of the polymer nanopost, *W*—width of the nanopost, *t*—thickness of the gold film, *a*—length of nanopost covered by gold, in case of partly covered nanoposts, and *b*—thickness of the gold film on the sides of the nanopost.

FDTD simulations of the optical spectra and electromagnetic field distributions were performed with FDTD Solutions (Lumerical Inc., Vancouver, BC, Canada) [34,35]. A full 3D setup with periodic boundary conditions was employed. The substrate and rectangular post is dielectric (*n* = 1.52), while the gold film on top is described by the Johnson and Christy coefficients, available in Lumerical’s material database. The refractive index of the environment was set to 1.33 or 1.38. Figure 2 depicts the simulation cell and involved geometrical parameters. The structure is illuminated by a plane wave from above.

## 3. Results

### 3.1. Morphology Analysis

**Figure 3:**
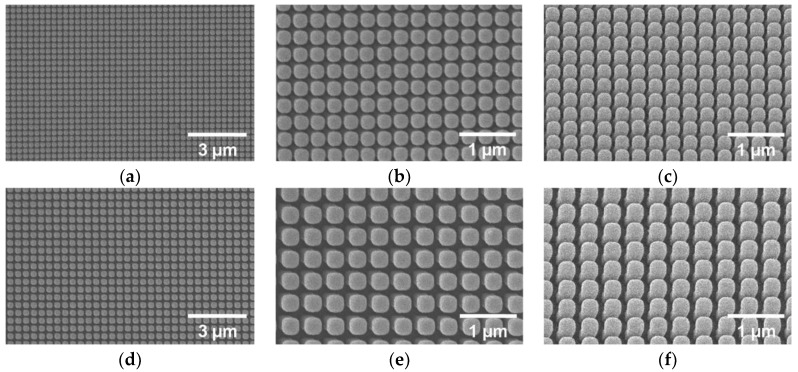
SEM images of the Au nanopost-shell arrays fabricated by NIL: (**a**–**c**) P300, and (**d**–**f**) P400 samples.

The SEM images of the Au NPSA fabricated by NIL are shown in Figure 3 and reveal that a large nanopatterned area has been fabricated with high order and uniformity. The array fabrication with the optimized NIL process parameters is highly reproducible and the NIL stamps are reused without array quality loss. SEM was used to study the topography of gold nanopost-shell arrays. As it can be observed by analyzing images in Figure 3, the width of the gold coated nanoposts is about 250 nm and 310 nm for the P300 and P400 samples, respectively. The period of the arrays are very close to 300 and 400 nm, in agreement with the specifications of the original molds used for NIL. The fabricated arrays are quite homogeneous and have very few defects, such as inclined nanoposts.

### 3.2. Optical Response of Au Nanopost-Shell Arrays

**Figure 4:**
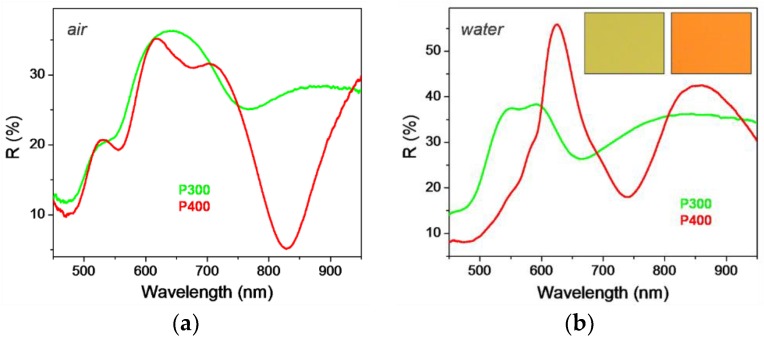
Optical reflectivity spectra of nanopost arrays: (**a**) in air and (**b**) in water. The insets show optical images of the nanopost arrays having the lattice parameter *P* as indicated on the graphs.

The optical response of the NPSA was first determined in both air and water as surrounding medium. The reflectivity spectra are displayed on Figure 4, and present several minima and maxima in the analyzed spectral domain. One can notice that by changing the medium from air to water the spectra are modified drastically, as expected from a plasmonic crystal exhibiting plasmon resonances in this range. In practice, such plasmonic crystals are often immersed in liquids, from which certain analytes need to be detected, or their refractive index needs to be monitored. Therefore, from this point onwards, all the spectra presented and discussed will be for samples immersed in water (or water–glycerol mixture), since it describes a situation pertaining to sensing, e.g., in microfluidics. The spectra recorded in water are somewhat similar in shape for the two NPSA studied, P300 and P400, both displaying a maximum and a minimum in the 500–850 nm region (Figure 4b). We note that this spectral region is of practical interest since it can be easily accessed with common optical components and spectrophotometers. The P300 array displays a broad *R* maximum in the region 512–625 nm, followed by a minimum around 665 nm. The spectrum of the P400 array displays a band peaking at 625 nm and a dip with the minimum at 740 nm. As known from previous studies, the dips in the reflectivity spectra are the spectrum features most relevant to sensing with plasmonic crystals comprising relatively thick gold films. These reflectivity dips indicate absorption due to excitation of surface plasmons, which are sensitive to changes of the adjacent medium. The different spectral properties of the two arrays can be observed even visually, since these display distinct colours, as it can be seen in the white light reflected images (insets in Figure 4b).

### 3.3. Optical Sensing with Au Nanopost-Shell Arrays

**Figure 5:**
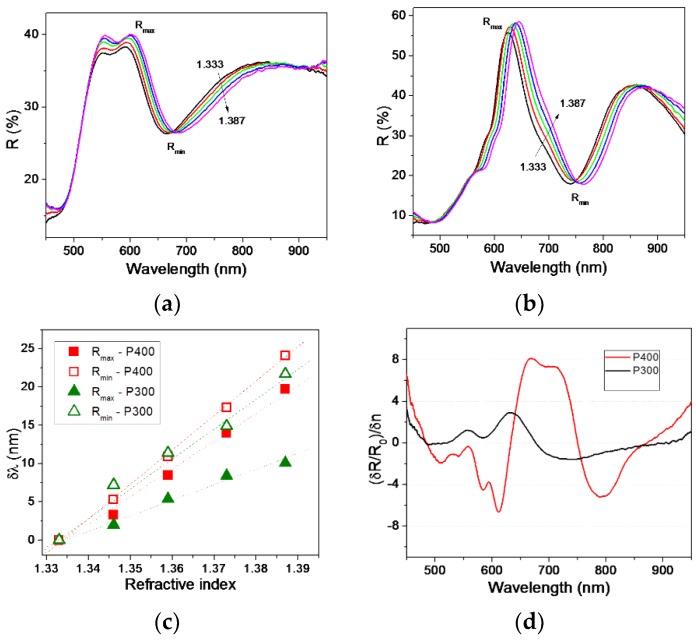
Reflectivity spectra of (**a**) array having *P* = 300 nm, and (**b**) array with *P* = 400 nm, in liquids of different refractive index. (**c**) Dependence of the shift of different reflectivity maxima/minima on refractive index. (**d**) Relative reflectivity variation per refractive index unit for the two studied nanopost-shell arrays.

The sensing capabilities of the fabricated NPSA were assessed by measuring their reflectivity spectra in media of different refractive index obtained by water–glycerol mixtures. The spectra recorded for the P300 and P400 nanopost shell arrays are presented on Figure 5a,b, respectively. The general trend observed is that the *R* minima shift towards the red part of the spectrum. The positions of reflectivity minima and maxima as a function of refractive index are displayed in Figure 5c, and exhibit a linear dependence.

One parameter used in the characterization of sensor performance is the sensitivity *S*, expressed as *S* = δλ_res_/δ*n*. From our measurements, we estimated values of sensitivity *S* around 408 nm/RIU (22 nm redshift) and 445 nm/RIU (24 nm redshift) for arrays P3 and P4, respectively, by considering the refractive index change from 1.333 to 1.387 (δ*n* = 0.054). These *S* values are very good ones among results available in the literature [36,37]. However, in practical implementations of sensing equipment, the shift of the reflectivity minimum can be detected as a relative reflectivity change δ*R*/*R*0 at a fixed wavelength. Furthermore, δ*R*/*R*_0_ can take different values strongly depending on the magnitude of the refractive index variation. We therefore propose to analyze here the parameter *S_R/n_* = (δ*R*/*R*_0_)/δ*n*, which represents a relative reflectivity variation per refractive index unit. Please note that the dependence of *S_R/n_* on wavelength can be obtained for any plasmonic systems, and different results can be easier compared. By analyzing this plot, one can also determine the wavelength at which the sensor would be most efficient if it worked by monitoring the intensity at a fixed wavelength. 

As can be observed in Figure 5d, high values of *S_R/n_* are obtained at spectral regions around the reflectivity minima, for both P300 and P400 NPSA.

In the next sections, we present results of FDTD simulations which were employed in order to understand the optical spectra of NSPA in water. We then focus on the sensing behavior of the NPSA, by investigating the role of different geometrical parameters on the sensing efficiency, aiming to identify the parameters which allow for sensor optimization.

### 3.4. FDTD Simulations of the Optical Response

**Figure 6:**
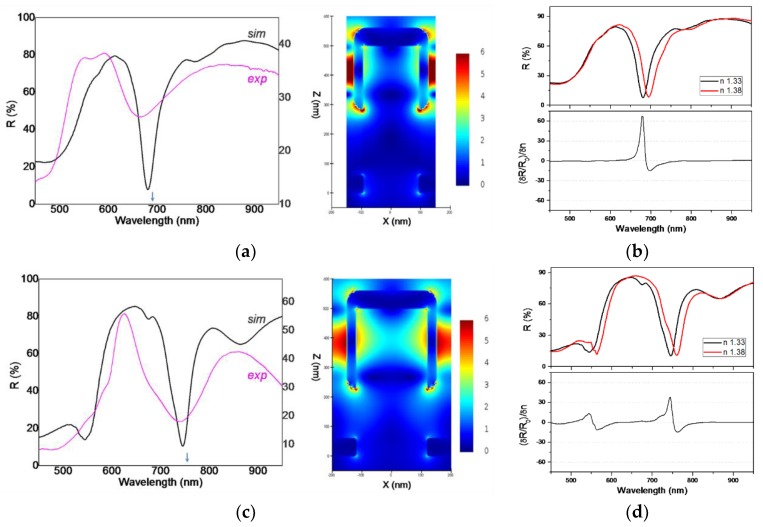
(**a**,**c**) Simulated spectra in water (thick black lines), and map of the electric field magnitude in the XZ plane, at the wavelength of *R* minimum (indicated by arrows), for arrays with *P* = 300 nm and *P* = 400 nm, respectively. Thin magenta lines are experimental spectra, presented for comparison. (**b**,**d**) Simulated spectra in media with different refractive index (top), and relative reflectivity variation per refractive index unit (bottom), for the two cases in (a,c).

We first explore the parameter space by simulating several geometries in which the parameters *P*, *W*, *t*, *a*, *b* (see Figure 2) were varied in the range suggested by SEM images, trying to find the best matching to the experiments. Figure 6a presents the simulated *R* spectra obtained with parameters *P* = 300 nm, *W* = 190 nm, *t* = 60 nm, *a* = 220 nm, *b* = *30* nm, which reproduce satisfactorily the spectrum measured on sample P300 (thin grey line in Figure 6a) in terms of shape and positions. Similarly, Figure 6c presents the simulated *R* spectrum obtained with parameters *P* = 400 nm, *W* = 240 nm, *t* = 60 nm, *a* = 275 nm, *b* = *30* nm, which match well the experimental spectrum on sample P400.

Obviously, the *R* minima in the simulated spectra are considerably narrower, which is to be expected due to the small variations in array period and nanopost width and shape. Such imperfections are inevitably present in real samples and act to broaden any resonances of the array, e.g., the *R* minimum. Next we analyzed the electric field distribution around the plasmonic nanostructures, which are displayed on the right part of Figure 6a,c for the two geometries, at the wavelength of *R* minimum. In both cases the electric field has a similar distribution, with a large region of enhanced fields at the lateral sides of the nanopost shell. This region extends in space to the neighbor nanopost shell, creating a large volume useful for sensing. Additionally, some field enhancement regions exist also at the lower edge of the nanoshell and at the top corners. The gold film at the base of the nanoposts appears to have a minor influence. In fact a separate simulation, in which the film at the bottom was removed from the setup, fully supports this statement. 

By using the same simulation setup, we then increased the refractive index of the medium from *n* = 1.33 to *n* = 1.38. From the obtained *R* spectra we then calculated the parameter *S_R/n_*. Results are presented in Figure 6b,d for the two geometries with *P* = 300 nm and *P* = 400 nm, respectively. Simulated values of *S_R/n_* are almost an order of magnitude higher than experimental ones; this was somewhat expected, having in mind the much sharper resonances (*R* dips) in the simulations. The simulations also confirmed the fact that the maximum sensitivity is obtained at the wavelength of *R* minima.

We can therefore conclude that the usefulness of the *R* dips for sensing are due to the excitation of surface plasmons at these wavelengths, and especially the spatial configuration of electric fields associated to these plasmon modes. These modes extend in the space between neighboring gold nanopost shells, and provide a large volume for probing nearby refractive index modifications.

### 3.5. Optimizing the Sensitivity by FDTD Simulations

**Figure 7:**
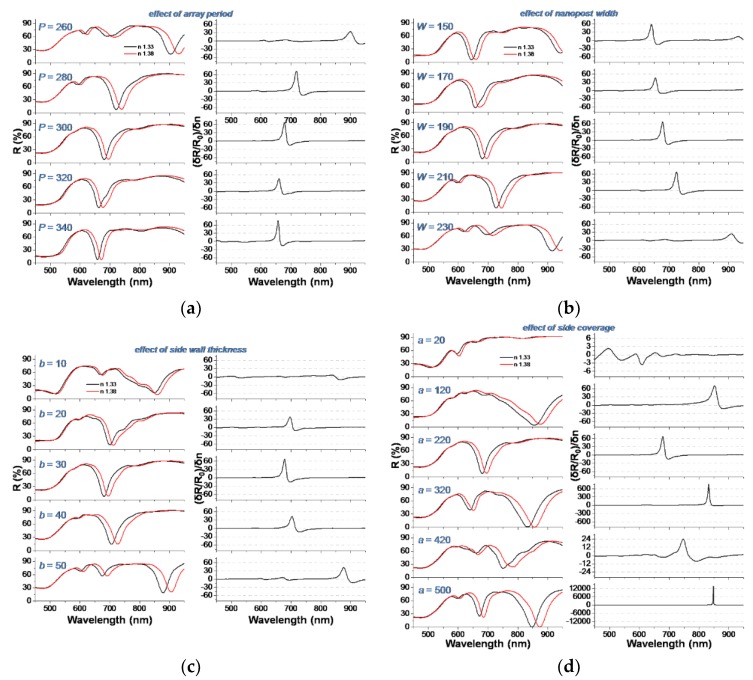
Dependence of the relative reflectivity variation per refractive index unit on several geometrical parameters of the nanopost-shell arrays: (**a**) array period *P*; (**b**) nanopost width *W*; (**c**) lateral coverage *a* of the nanopost by gold film; (**d**) lateral thickness *b* of the gold film on nanoposts.

In this section we systematically explore the role of several geometrical parameters of the NPSA on the sensitivity *S_R/n_*. We performed different sets of simulations in which the influence of array period *P*, nanopost width *W*, gold film thickness on the nanopost sides *b*, and coverage of the nanoposts’ sides *a* (see Figure 2) were investigated one at a time. First the array period *P* was varied from 260 to 340 nm, while keeping *W* = 190 nm, *t* = 60 nm, *a* = 220 nm, and *b* = 30 nm. Reflectivity spectra for *n* = 1.33 and *n* = 1.38, together with the sensitivity *S_R/n_* are presented in Figure 7a. The main effect of modifying the period in this range was to modify the spectral position at which the maximum sensitivity is obtained. It can be observed that by decreasing *P*, the interactions between neighboring nanopost shells become stronger, leading to a large redshift. The magnitude of the sensitivity can also be increased by selecting a *P* value which produces a narrow and deep reflectivity dip, e.g., *P* = 340 nm in our case.

Next, the nanopost width *W* was varied from 150 to 230 nm, while keeping *P* = 300 nm, *t* = 60 nm, *a* = 220 nm, and *b* = 30 nm. Reflectivity spectra and the sensitivity *S_R/n_* are presented in Figure 7b. Increasing the nanopost width has the effect of gradually shifting the *R* dip towards the red, since the size of the gold shell increases. For *W* = 230 nm, a much larger shift is observed, which is due to the fact that the neighbor nanoposts are very close to each other, thus their plasmonic coupling becomes stronger. This coupling has also the effect of broadening the *R* dips (observed also for *P* = 260 in Figure 7a), which in turn decreases the sensitivity *S_R/n_*. Again, the spectral position and magnitude of the maximum sensitivity can be adjusted, although the effect on magnitude is not spectacular.

Another parameter that was varied in order to understand its role on sensitivity is the thickness of the gold film on the sides of the nanopost *b*, i.e., side thickness of the gold shell. Results in Figure 7c indicate that, again, the position of *R* dips and sensitivity maxima can be controlled by side thickness. However, the dependence is more complex in this case, as the *R* spectra present different shapes, e.g., for the lowest *b* values the dips are broad and irregular, with multiple shoulders.

The degree of side coverage of the nanoposts by gold, expressed by parameter *a* (see Figure 2) is the last parameter varied, in the range 20–500 nm, corresponding to a laterally non-coated to a fully coated nanopost. The other parameters kept fixed in this set of simulations are *P* = 300 nm, *W* = 190 nm, *t* = 60 nm, *a* = 220 nm, and *b* = 30 nm. The results, presented on Figure 7d, are surprising, by the fact that side coverage appears to influence both the reflectivity spectra and the obtained sensitivity much more drastically that the other studied parameters. By increasing *a* from 20 to 500 nm (full coverage) the reflectivity spectra change from having no useful reflectivity dip in the studied spectral range, to having dips of different width and depth. For *a* = 20 nm, a very low sensitivity *S_R/n_* in the range 2–3 is obtained; increasing *a* to 120 and 220 nm yields peak *S_R/n_* values in the range 60–70 RIU^−1^, as already observed in many cases discussed above; further increasing to 320 nm produces a strong increase, *S_R/n_* reaching the range 700–800 RIU^−1^, while for *a* = 420 nm only modest values of *S_R/n_* in the range 20–30 RIU^−1^ are obtained. For the fully covered nanoposts, an exceptionally high value of *S_R/n_* approaching 14,000 RIU^−1^ was obtained, which is due to the extreme depth of the reflectivity dips in this case. Although we chose not to discuss and base our analysis on the more frequently encountered sensitivity *S* = δλ_res_/δ*n*, this takes values in the range 300–660 nm/RIU for the different configuration simulated, in the same range as the experimental results.

## 4. Discussion

Very often, NIL patterns are fabricated in a polymer layer on top of another solid substrate [21]. In other approaches, the nanoposts (named also nanopillars by some authors) are made of silicon, which is not flexible, and imposes some limits on the applications [38]. Interference lithography followed by ion beam milling was proposed by other authors, which yielded nanoposts with lower aspect ratios, and rather poorly defined spectral features [39]. Monolithic nanoposts imprinted directly into substrate were also demonstrated, made by using nanoporous alumina as the NIL stamp, which does not yield ordered arrays of nanoposts [26,40]. Finally, in some of these examples, metal-coated nanoposts were exploited for their SERS capability, and not for SPR sensing. 

In this work, gold nanopost-shell arrays were fabricated by gold sputtering over monolithic arrays of dielectric nanoposts fabricated by NIL. One important aspect is that a NIL process able to directly imprint nanoposts onto a flexible polymer substrate was implemented. Albeit being rather broad, the reflectivity dips exhibited by the fabricated plasmonic crystals provide the means for obtaining very good sensing behavior, with a sensitivity in the range of large, above-average reported sensitivities. 

As demonstrated by the simulation results presented above, by tuning several geometrical parameters of the gold nanopost-shell arrays, one can design sensors working efficiently at the desired wavelength. Furthermore, all studied parameters have an effect on the sensitivity of the plasmonic sensor, with the most drastic effect induced by the degree of side coverage. Our results indicate that nanoposts that are fully coated by the gold film have the potential for achieving unusually high sensitivities. Their reflectivity approach zero at the minimum, therefore any minute change produces a large relative change, which can be efficiently monitored. From a practical point of view, array period *P* and nanopost width *W* can be easily controlled by the fabrication of the stamps for NIL. On the other hand, the lateral thickness and degree of coverage, which are more important for increasing the sensitivity, are the parameters more challenging to control experimentally. The problem could be tackled by selecting the proper deposition methods among thermal or electron-beam evaporation, magnetron sputtering, pulsed laser deposition, or even chemical methods such as chemical grafting coupled to electroless plating. For example, by evaporation, which provides a highly directional deposition, by sufficiently decreasing the *W*/*P* ratio (i.e., increasing space between nanoposts), and slightly tilting the nanopost array and rotating it around an axis normal to its surface, conditions for avoiding shadowing effects and obtaining full nanopost coverage could be realized. However, a larger distance between posts could affect the reflectivity minima, which might become shallower due to the lower density of nanopost shells, negatively affecting the sensitivity. It is therefore a future task to find a good compromise for fulfilling the conditions which can lead to ultrahigh sensitivity plasmonic sensors based on metal-fully-coated dielectric nanopost arrays.

## 5. Conclusions

Nanoimprint lithography was applied to fabricate monolithic polymer nanopost arrays, which were subsequently sputter-coated by gold films. The obtained plasmonic crystal presents optical reflectivity dips, which are attributed to excitation of surface plasmons. The electromagnetic field distribution associated to these modes exhibit enhanced fields mainly on the sides of the nanopost-shells. The spatial extension of these plasmon modes provides the means for an efficient sensing of refractive index changes in the embedding medium. By FDTD simulations, we have shown how the array period, nanopost width, side shell thickness, and degree of side coverage can influence the reflectivity spectra and the sensing efficiency of these plasmonic nanopost-shell arrays. A particularly huge sensitivity was obtained for fully-coated nanoposts, in which reflectivity minima approach zero. Besides the sensitivity optimization, the systematic simulations presented in Section 3.5 are also useful for a better understanding of the plasmonic properties of these nanopost-shell arrays. By pinpointing the role of each parameter on the *R* spectrum, relevant information on the plasmon modes involved can be deduced.

These results can serve as guidelines for the future design and optimization of flexible, tunable, cost-efficient, and highly sensitive surface plasmon resonance sensors. Besides, the gained knowledge on the nanopost-shell arrays plasmon resonances can be exploited on a more general level for, e.g., for developing plasmonic filters or absorbers, and substrates for surface-enhanced spectroscopies.
